# Decreased N-TAF1 expression in X-linked dystonia-parkinsonism patient-specific neural stem cells

**DOI:** 10.1242/dmm.022590

**Published:** 2016-04-01

**Authors:** Naoto Ito, William T. Hendriks, Jyotsna Dhakal, Christine A. Vaine, Christina Liu, David Shin, Kyle Shin, Noriko Wakabayashi-Ito, Marisela Dy, Trisha Multhaupt-Buell, Nutan Sharma, Xandra O. Breakefield, D. Cristopher Bragg

**Affiliations:** 1 The Collaborative Center for X-Linked Dystonia-Parkinsonism, Department of Neurology, Massachusetts General Hospital, Charlestown, MA 02129, USA; 2 Harvard Brain Science Initiative, Harvard Medical School, Boston, MA 02114, USA; 3 Center for Molecular Imaging Research, Department of Radiology, Massachusetts General Hospital, Charlestown, MA 02129, USA

**Keywords:** X-linked dystonia-parkinsonism, Induced pluripotent stem cells, TAF1

## Abstract

X-linked dystonia-parkinsonism (XDP) is a hereditary neurodegenerative disorder involving a progressive loss of striatal medium spiny neurons. The mechanisms underlying neurodegeneration are not known, in part because there have been few cellular models available for studying the disease. The XDP haplotype consists of multiple sequence variations in a region of the X chromosome containing *TAF1*, a large gene with at least 38 exons, and a multiple transcript system (MTS) composed of five unconventional exons. A previous study identified an XDP-specific insertion of a SINE-VNTR-Alu (SVA)-type retrotransposon in intron 32 of *TAF1*, as well as a neural-specific TAF1 isoform, N-TAF1, which showed decreased expression in post-mortem XDP brain compared with control tissue. Here, we generated XDP patient and control fibroblasts and induced pluripotent stem cells (iPSCs) in order to further probe cellular defects associated with this disease. As initial validation of the model, we compared expression of *TAF1* and MTS transcripts in XDP versus control fibroblasts and iPSC-derived neural stem cells (NSCs). Compared with control cells, XDP fibroblasts exhibited decreased expression of *TAF1* transcript fragments derived from exons 32-36, a region spanning the SVA insertion site. N-TAF1, which incorporates an alternative exon (exon 34′), was not expressed in fibroblasts, but was detectable in iPSC-differentiated NSCs at levels that were ∼threefold lower in XDP cells than in controls. These results support the previous findings that N-TAF1 expression is impaired in XDP, but additionally indicate that this aberrant transcription might occur in neural cells at relatively early stages of development that precede neurodegeneration.

## Abstract (precis)

**Summary:** This study describes a new iPSC model of X-linked dystonia-parkinsonism (XDP), which was initially validated by demonstrating a similar transcriptional defect as has been previously reported in XDP brain tissue.

## INTRODUCTION

The dystonias are a diverse collection of movement disorders arising from different etiologies that lead to characteristic patterns of involuntary muscle contractions and twisted postures (Albanese et al., 2013a). Most cases in which dystonia is the primary clinical manifestation seem to have a genetic predisposition, with over 25 gene loci linked to different forms of the disease (Lohmann and Klein, 2013). Among the many subtypes of dystonia are numerous examples that combine clinical features of Parkinson's disease (PD), such as (1) DOPA-responsive dystonia, caused by variations in genes encoding dopamine biosynthetic enzymes (*GCH1*, *TH*, *SR*; Lee and Jeon, 2014); (2) rapid-onset dystonia-parkinsonism (RDP), caused by variations in a subunit of the sodium/potassium ATPase (*ATP1A3*; Geyer and Bressman, 2011); (3) DYT16 dystonia, associated with variations in the PKR regulatory protein, PACT (*PRKRA*; Zech et al., 2014; Camargos et al., 2012); and (4) other degenerative disorders such as Wilson's disease and neurodegeneration with brain iron accumulation (NBIA; Schneider et al., 2009; Schneider and Bhatia, 2010). Yet in no form of hereditary dystonia is a connection to PD more apparent than in X-linked dystonia-parkinsonism (XDP, DYT3, OMIM #3142590), a progressive neurodegenerative disease endemic to the island of Panay, Philippines (Lee et al., 2011). XDP follows an unusual clinical course, consisting primarily of dystonic symptoms at early disease stages that shift over time towards a more parkinsonian phenotype (Lee et al., 2002, 2011). The underlying disease mechanisms and pathogenic substrates are not clear, but neuropathological studies indicate that XDP involves a progressive loss of medium spiny neurons in the striatum (Waters et al., 1993; Goto et al., 2005) and decreased numbers of neural progenitor cells within the subventricular zone (Goto et al., 2013).

Previous genetic linkage (Wilhelmsen et al., 1991; Kupke et al., 1992; Graeber and Müller, 1992) and association (Müller et al., 1994; Haberhausen et al., 1995; Németh et al., 1999) studies mapped the XDP founder haplotype to a region of chromosome Xq13.1, that was recently narrowed further to an interval of ∼294 kb (Domingo et al., 2015). The region contains five known genes, *NONO*, *TAF1*, *OGT*, *ACRC* and *CXCR3*, as well as an intergenic multiple transcript system (MTS) composed of five unconventional exons (Nolte et al., 2003; Herzfeld et al., 2007; Domingo et al., 2015). Sequencing of this region revealed seven XDP-specific sequence variants: five disease-specific single nucleotide changes (designated DSC1, 2, 3, 10 and 12), a 48-bp deletion, and a SINE-VNTR-Alu (SVA)-type retrotransposon insertion (Nolte et al., 2003; Makino et al., 2007). Only one variant, DSC3, falls within a potential coding region, introducing a single nucleotide substitution in one of the MTS exons (Nolte et al., 2003). The other variants are localized either within *TAF1* introns or the intergenic region containing the MTS that is 3′ to exon 38 of *TAF1*. To date it has not been possible to exclude any of these variants as pathogenic, as extensive genotyping of large numbers of XDP individuals and ethnically matched control subjects has detected all markers in essentially all cases; the few exceptions, which were negative for all markers, were considered likely phenocopies (Domingo et al., 2015).

Of the five known genes within this interval, *TAF1* has arguably received the most attention as the one likely to be responsible for XDP pathogenesis. *TAF1* is a large gene with at least 38 exons that encodes a transcription factor, TATA-binding protein-associated factor-1 (TAF1), which is part of the transcription factor IID (TFIID) complex involved in RNA polymerase II-mediated transcription (Ruppert et al., 1993; Thomas and Chiang, 2006). The hypothesis that *TAF1* might be the culprit in XDP has been based in part on the relationship between the XDP sequence variants and its genomic structure and transcripts. Three of the haplotype markers (DSC10, DSC12 and the SVA) alter sequences within *TAF1* introns, and RNAs derived from MTS exons can reportedly splice to various *TAF1* transcripts (Nolte et al., 2003; Herzfeld et al., 2007; Makino et al., 2007). In addition, a previous study identified a neural-specific TAF1 isoform, N-TAF1, which was significantly downregulated in XDP caudate relative to control brain tissue (Makino et al., 2007). N-TAF1 contains an alternative exon, exon 34′, of only six nucleotides, which adds an alanine and lysine to the encoded protein (Makino et al., 2007; Sako et al., 2011; Jambaldorj et al., 2012). It has been suggested that the decreased expression of N-TAF1 in XDP might be the result of the 2.6 kb SVA insertion within intron 32 of *TAF1* (Makino et al., 2007). However, it is not clear how this insertion acts to interfere with transcription of N-TAF1.

In this study we generated XDP and matched control induced pluripotent stem cell (iPSC) lines that can be differentiated into neural cells in order to establish a model system for probing cellular defects associated with the disease. Towards that end, we derived fibroblasts from confirmed XDP individuals and unaffected family members as matched control subjects, reprogrammed these cells to iPSCs, and then re-differentiated iPSCs to generate XDP and control neural stem cells (NSCs). As a first application of this model, we assayed expression of *TAF1* and MTS*-*derived transcripts in both the iPSC-derived NSCs as well as the parent fibroblast lines. These analyses revealed genotypic differences in the expression of multiple *TAF1* transcripts in fibroblasts and decreased expression of N-TAF1 in XDP versus control NSCs. These findings are consistent with the previous report of an XDP-related defect in N-TAF1 expression (Makino et al., 2007), indicating that the patient iPSC-derived neural cells recapitulate a phenotype detected in patient post-mortem brain tissue. In addition, the present results further suggest that this defect might not be solely associated with advanced disease stages but might instead occur in XDP neural cells at early stages that precede the development of neuronal cell death.

## RESULTS

### Clinical characteristics and genotype of XDP individuals

Of the five XDP individuals that participated in this study, only one (32517; Table 1) was examined prior to undergoing deep brain stimulation (DBS). At the time of fibroblast derivation, this subject presented with segmental dystonia affecting the jaw and larynx. Dystonic symptoms subsequently worsened and generalized to other muscle groups over the course of 1 year, at which point the individual underwent bilateral pallidal DBS. The other four subjects had similar clinical histories, involving initial onset of dystonic symptoms in various muscle groups. At the time of skin biopsy, these subjects exhibited variable degrees of dystonia, ranging from generalized symptoms affecting the left hand, torso and left leg in one subject (34363) to focal neck dystonia in another (33109). Bradykinesia without rigidity was a common feature among subjects. All control family members showed no neurological abnormalities upon examination.

**Table 1.**
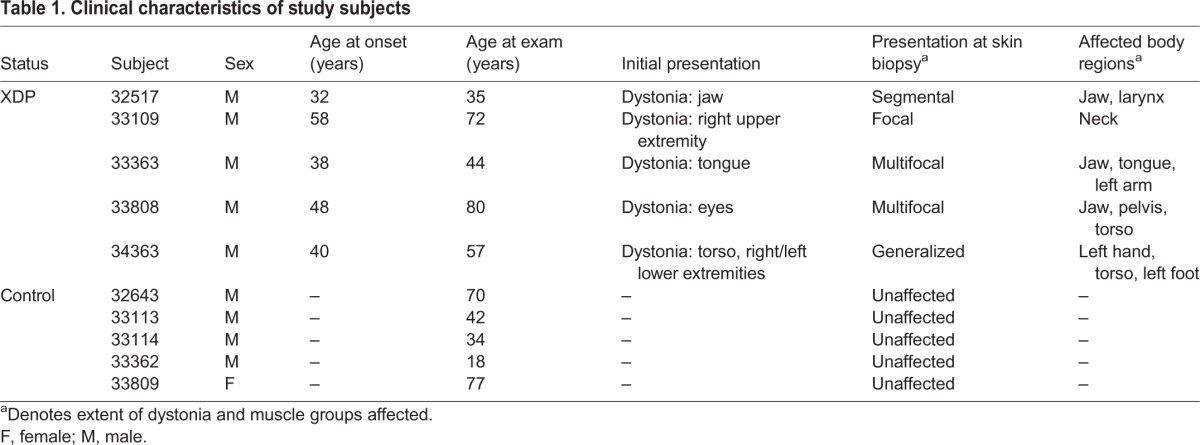
**Clinical characteristics of study subjects**

**Fig. 1.**
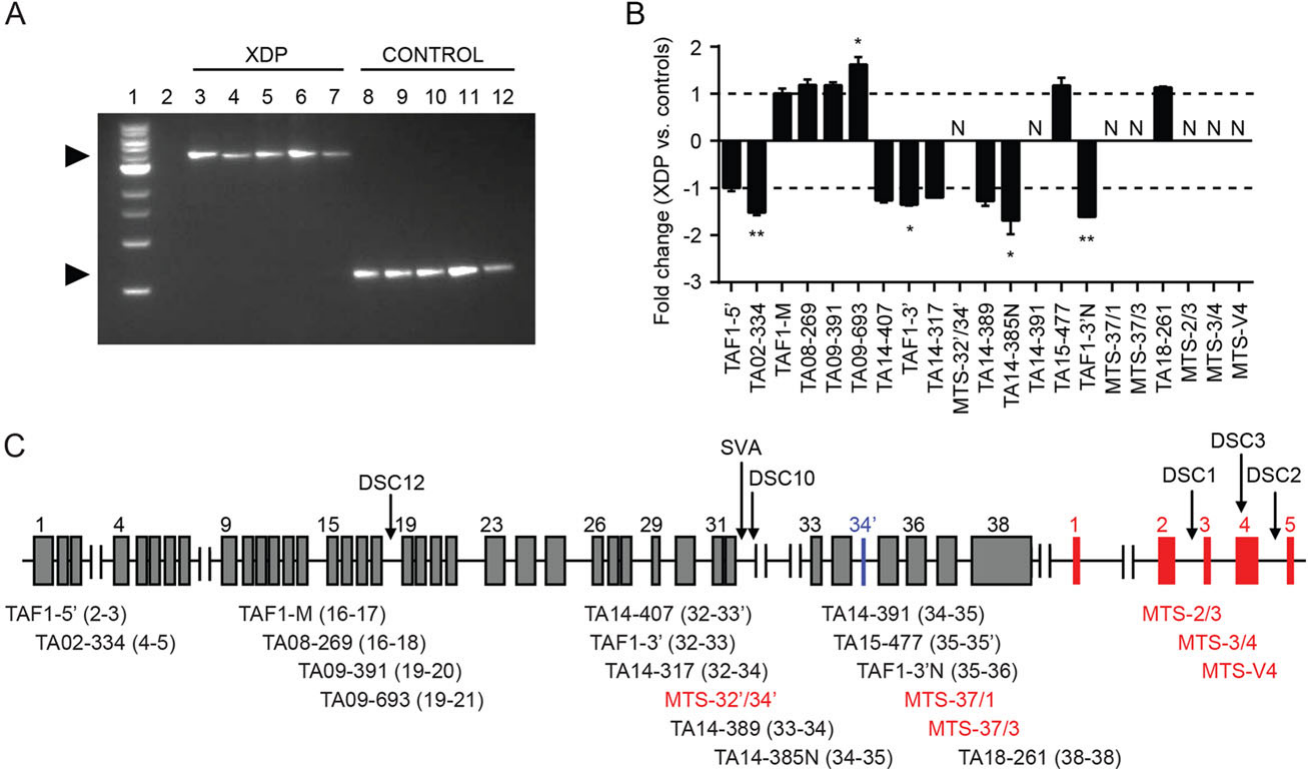
***TAF1* and MTS transcript expression levels in fibroblasts.** (A) Genomic DNA (gDNA) from all individuals was PCR amplified with primers flanking the insertion site to confirm the presence of the SVA. Lane 1: 1 kb DNA ladder. Lane 2: no template control (H_2_O). Lanes 3-7: XDP lines (left to right) 32517, 33109, 33363, 33808, 34363. Lanes 8-12: Control lines (left to right) 32643, 33113, 33114, 33809, 33362. The predicted 3229 bp SVA product was present in all XDP samples (upper arrow), whereas controls had a product of ∼599 bp (lower arrow), a difference consistent with the size of the SVA. (B) Quantitative expression analysis of *TAF1* transcript fragments in XDP vs control fibroblasts (*n*=5 each) based on comparative Ct method. Expression levels were normalized to the mean of housekeeping genes *HPRT1* and *TFRC*. Levels of transcript fragments amplified by primer sets TA02-334, TAF1-3′, TA14-385N and TAF1-3′N were significantly lower in XDP vs control cells, whereas expression of the transcript amplified by TA09-693 was significantly increased in XDP vs control samples. The neural-specific transcript, N-TAF1, amplified by primer set TA14-391, as well as all six transcripts incorporating MTS sequences, were not detected in fibroblasts. Data represent mean fold changes±standard errors, analyzed by Student's *t*-test. *P<0.05; **P<0.01; N, not detected. (C) Schematic depicting approximate position of the DSCs and SVA relative to *TAF1* (gray) and MTS exons (red). N-TAF1 incorporates the alternative exon, 34′ (blue). Below the graph are shown the relative positions of primer sets used for qRT-PCR. Numbers in parentheses indicate exons targeted by forward and reverse primers. Primers MTS-32′/34′, MTS-37/1 and MTS-37/3 amplify fragments in which the indicated *TAF1* exons are reportedly spliced to MTS exons.

Genotypes of affected individuals and control family members were determined by PCR for six of the seven reported haplotype markers (Nolte et al., 2003; Makino et al., 2007). The five single nucleotide substitutions (DSC1, 2, 3, 10 and 12) were evaluated by PCR amplification followed by Sanger sequencing of the products. The affected subjects showed a consistent pattern, with the same nucleotide substitutions at each DSC that had been previously associated with XDP (Table 2). The control individuals all had wild-type sequences at each site. The 2.6 kB SVA retrotransposon was detected by long-range PCR amplification using primers flanking the insertion site. A single PCR product was detected in both XDP and control samples, which differed in size by ∼2.6 kB (0.6 kb in control versus 3.2 kb in XDP samples), consistent with the size of the SVA (Fig. 1A). There were no genotypic differences noted in growth rate or cell division among the fibroblast lines. As expected, fibroblasts from the older donors, 33808 (XDP) and 33809 (control), had decreased doubling times in culture compared with the lines derived from younger subjects. However, most of the lines grew at comparable rates, and all fibroblast experiments were performed at equivalent early passages.

**Table 2.**
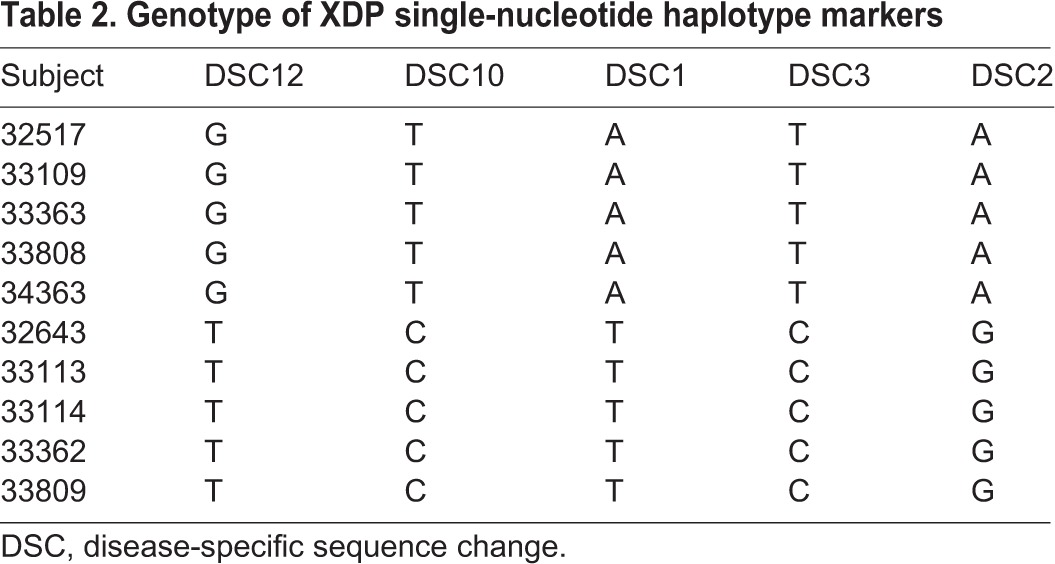
**Genotype of XDP single-nucleotide haplotype markers**

### Analysis of *TAF1* and MTS transcripts in fibroblasts

We compared expression of transcripts derived from *TAF1* and the putative MTS exons in XDP versus control fibroblasts using a panel of previously described primers (Makino et al., 2007) interrogating 21 mRNAs (Fig. 1B,C). Of the 21 transcripts assayed, seven were undetectable in both XDP and control fibroblast samples. The panel included six primer sets targeting transcripts that reportedly incorporate MTS sequences, either alone (MTS-2/3, MTS-3/4, MTS-V4) or spliced to exons of *TAF1* (MTS-32′/34′, MTS-37/1, MTS-37/3) (Makino et al., 2007). None of these mRNAs was expressed in fibroblasts. In addition, primer set TA14-391, which was previously shown to be specific to the neural TAF1 isoform, N-TAF1, did not detect a product in fibroblasts.

Among the remaining 14 mRNAs tested, five showed differential expression distinguishing XDP from control samples at *P*<0.05 (Fig. 1B). Three primer sets (TAF1-3′, TA14-385N and TAF1-3′N) amplified transcript fragments derived from exons 32-36 that were significantly decreased in XDP fibroblasts compared with control cells. These exons flank the SVA insertion in intron 32 and span the region containing the alternative exon, 34′, from which the neural isoform, N-TAF1, is generated. Other primers within the panel also targeted fragments derived from these exons; although levels of their respective products were also lower in XDP cells relative to controls, the differences did not achieve statistical significance. Two other transcript fragments showed significant differences in expression in XDP versus control cells: one fragment (amplified by TA02-334) derived from exons 4-5 had lower levels in XDP cells, and another (from TA09-693) incorporating sequences from exons 19-21 had higher expression in XDP cells than in controls. In addition to *TAF1* transcripts, we assayed expression of three genes also contained within the XDP genomic region (*CXCR3*, *ACRC* and *OGT*) but none showed differential expression in XDP versus control fibroblasts (Fig. S1).

In other genes, SVA insertions have been shown to generate aberrant transcripts consisting of retrotransposon sequences spliced to RNA derived from the surrounding exons (Hancks and Kazazian, 2012). To probe for such exonization of the SVA in *TAF1*, we designed two additional primer sets flanking the insertion (Table S1) and performed qRT-PCR on RNA from XDP and control fibroblasts. Because aberrant transcripts might potentially be targeted for nonsense-mediated decay (NMD), fibroblasts were cultured in a specific NMD inhibitor, NMDI14 (Martin et al., 2014), prior to RNA isolation and reverse transcription. Gel electrophoresis of the PCR products showed bands of equivalent sizes in XDP and control samples (Fig. S2), suggesting that SVA sequences were not incorporated into these particular transcript fragments.

The observation that N-TAF1 was not detectable in fibroblast RNA seems consistent with its previously documented pattern of neural tissue-specific expression (Makino et al., 2007; Sako et al., 2011; Jambaldorj et al., 2012). As additional validation, we used the TA14-391 primer set to assay N-TAF1 transcript levels in RNA obtained from SH-SY5Y human neuroblastoma cells, as well as human brain. Moderate N-TAF1 transcript expression was detected in SH-SY5Y cells, which increased slightly in cells differentiated in all-*trans*-retinoic acid (ATRA) for 72 h (Table 3). N-TAF1 expression in human brain was considerably higher, representing a more than 60-fold increase above the levels detected in undifferentiated SH-SY5Y cells.

**Table 3.**
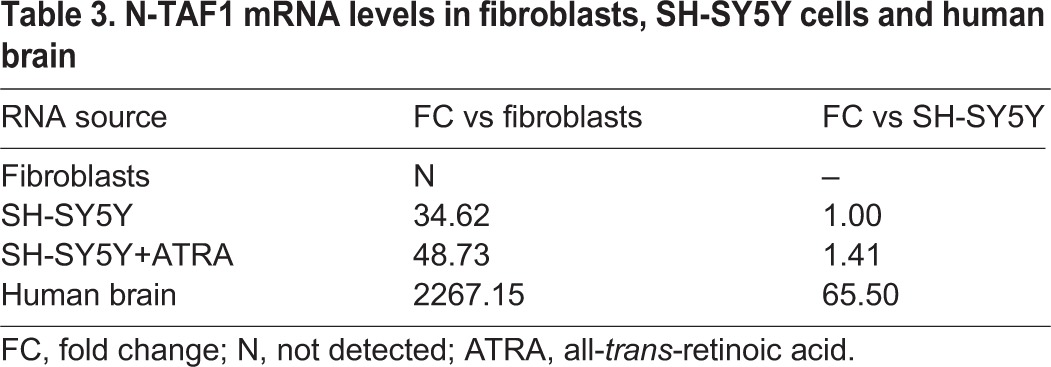
**N-TAF1 mRNA levels in fibroblasts, SH-SY5Y cells and human brain**

### Generation of iPSCs and neural conversion

**Fig. 2.**
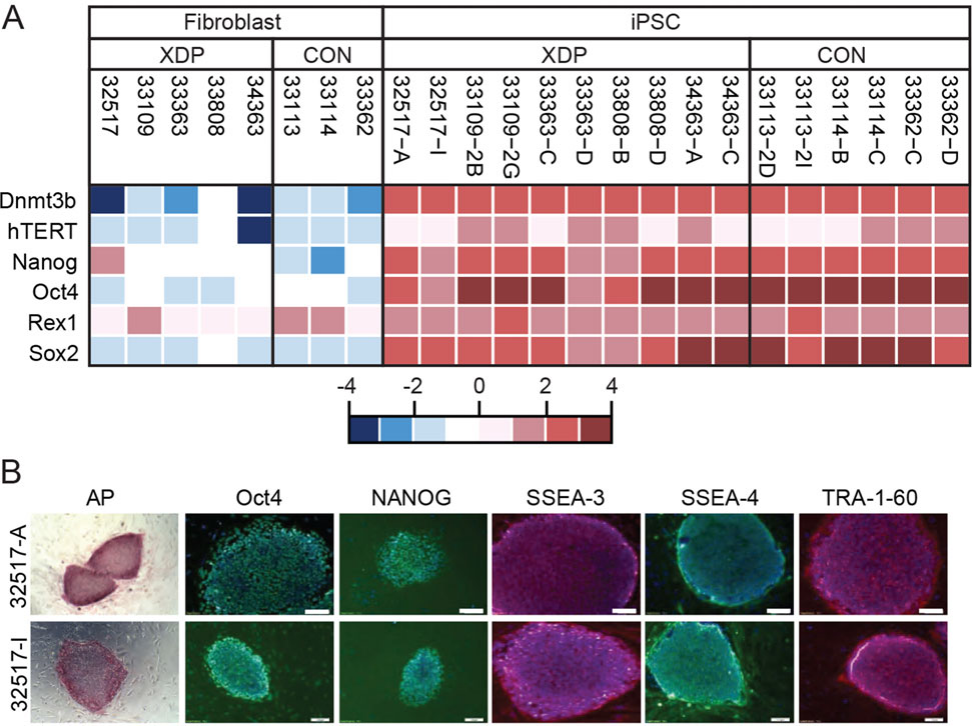
**Derivation of XDP and control induced pluripotent stem cells.** (A) Expression of pluripotency-related genes *Dmnt3b*, *hTERT*, *Nanog*, *Oct4*, *Rex1* and *Sox2* in each reprogrammed XDP and control iPSC clone compared with the parent fibroblast lines determined by qRT-PCR. Heatmap based on ΔCt values. Red, high expression; blue, low expression. (B) Pluripotency marker protein expression in two representative iPSC clones (A and I) derived from XDP fibroblast line 32517. Both clones expressed alkaline phosphatase (AP), Oct4, Nanog, SSEA-3, SSEA-4 and TRA-1-60. Immunostaining was visualized using secondary antibodies conjugated to Alexa Fluor 488 (green; Oct4, Nanog and SSEA-4) or Alexa Fluor 594 (red: SSEA-3 and Tra-1-60) with DAPI (blue) to visualize nuclei. Images shown represent overlays. Scale bars: 10 µm.

Given previous data specifically implicating N-TAF1 in XDP pathogenesis (Makino et al., 2007), a major objective of this study was to generate XDP cell lines in which expression of this variant could be further examined. Towards that end, we reprogrammed five XDP and four control fibroblast lines to iPSCs that could be re-differentiated into neural cells. Reprogramming was accomplished using non-integrating Sendai virus. Control fibroblast line 33809 exhibited slow growth in culture and typically underwent senescence at lower passages than the other cell lines, making it difficult to expand sufficiently for viral inoculation. Given that fibroblast growth rate can greatly influence reprogramming efficiency (Hanna et al., 2010), this cell line was not reprogrammed. All of the XDP fibroblasts and three of the control lines (33113, 33114 and 33362) generated at least two iPSC clones each that exhibited significant upregulation of pluripotency-related genes, *Dmnt3b*, *hTERT*, *Nanog*, *Oct4*, *Rex1* and *Sox2*, compared with the parent fibroblast lines, as measured by qRT-PCR (Fig. 2A). Clones also expressed alkaline phosphatase and were immunopositive for standard iPSC markers Oct4, Nanog, stage specific antigen (SSEA)-3 and SSEA-4, and TRA-1-60 (Fig. 2B; Figs S3, S4). Control fibroblast line 32643 was reprogrammed but all iPSC clones sampled contained different chromosomal abnormalities, even though the parent fibroblast line was karyotypically normal. These clones were therefore not characterized. All other iPSC clones retained normal karyotypes (Fig. S5).

**Fig. 3.**
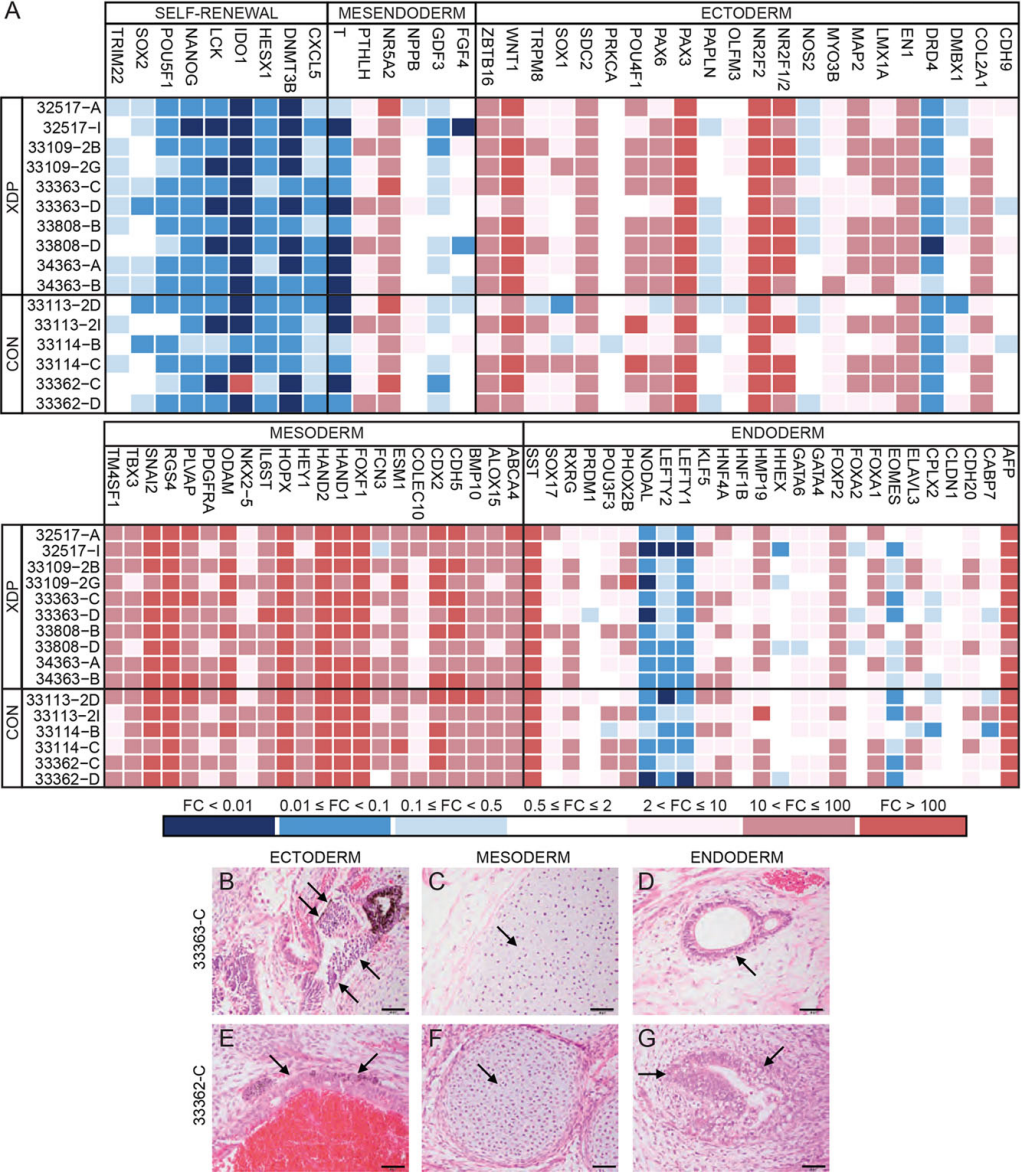
**Pluripotency analyses.** (A) Trilineage potential of each XDP and iPSC clone determined by Taqman Scorecard Assay analysis of RNA from embryoid bodies differentiated in culture from each cell line. Heatmaps depicting expression of 94 marker genes related to self-renewal, primitive mesendoderm, mature ectoderm, mesoderm and endoderm. Values represent mean fold changes (FC) for each gene relative to Scorecard reference pluripotent cell lines, calculated by PSC Scorecard analysis software. Red, high expression; blue, low expression. (B-G) Representative XDP (33363-C; upper panel) and control (33362-C; lower panel) teratomas produced in mice following cell implantation. Arrows denote ectodermal neuroepithelium (B) and hair follicle cells (E); mesodermal cartilage (C,F); endodermal mucous gland (D) and gut epithelium (G). Scale bars: 100 µm.

**Fig. 4.**
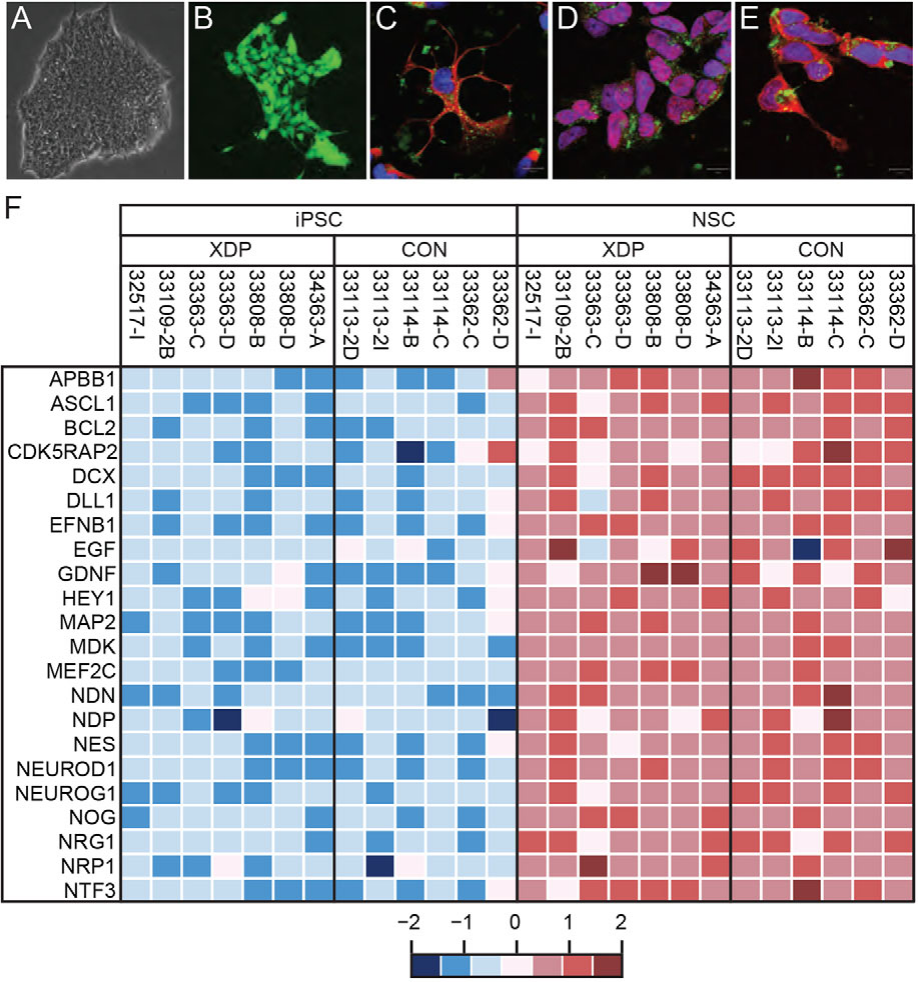
**Generation and characterization of neural stem cells.** (A) Brightfield micrograph depicting characteristic morphology of a representative iPSC colony on Geltrex prior to neural conversion. (B) Epifluorescent image of neural stem cells (NSCs) generated by conversion of iPSCs in PSC Neural Induction Medium, labeled with calcein-AM (Life Technologies). NSCs on Geltrex formed discrete clusters of cells with occasional fine processes. Images in A and B captured at final magnification of 20×. (C-E) NSCs were also evaluated by immunofluorescence for expression of (C) nestin; (D) Sox1; and (E) musashi. Staining for each target was detected by a secondary antibody coupled to Alexa Fluor 594 (red), along with counterstaining to visualize cytoplasm (Wheat germ agglutinin-Alexa Fluor 488; green) and nuclei (Topro-3-iodide; blue). Images represent overlays of all three channels captured by laser confocal microscopy at final magnification of 100×. Immunoreactivities for nestin and musashi seem predominantly cytoplasmic, whereas Sox1 labeling was observed primarily within the nucleus. Scale bars: 10 µm. (F) Heatmap of ΔCt values depicting comparative expression of 22 neural marker genes in XDP and control NSCs vs corresponding iPSCs from which they were differentiated. Red, high expression; blue, low expression.

To quantify functional pluripotency of each iPSC line, embryoid bodies (EBs) were generated in culture from all clones and assayed for expression of germ layer marker genes using the Taqman hPSC Scorecard Assay (Bock et al., 2011; Tsankov et al., 2015). As expected, the differentiated EBs exhibited little to no expression of genes involved in self-renewal but were positive for multiple markers of each lineage (Fig. 3A). There was some variability in expression of individual genes across the various clones, but the overall pattern was largely consistent and revealed no genotypic differences in pluripotency. Mesoderm-related genes seemed to be more highly expressed than markers of endoderm and ectoderm, but the observed signatures suggest that all clones retain the capacity to differentiate towards all three lineages. In parallel to the EB analysis, selected iPSC clones were also injected into mice to evaluate pluripotency *in vivo*. All clones tested formed teratomas containing cells representative of the three germ layers, based on morphology visible by Hematoxylin and Eosin (H&E) staining (Fig. 3B-G). The iPSC lines had comparable morphologies (Fig. 4A) and growth behavior in culture, except for clone 33109-2G, which exhibited a high rate of spontaneous differentiation that interfered with propagation and prevented neural conversion. This behavior seemed to be a clone-specific property, as the companion line, 33109-2B, did not differ in its growth characteristics from the other iPSC lines. All iPSC clones have been deposited at the repository at WiCell (Madison, WI, USA; www.wicell.org) and are publicly available.

To generate NSCs, XDP and control iPSCs were cultured in PSC Neural Induction Medium for seven days, at which point they were re-plated on Geltrex-coated culture dishes and propagated in Neural Expansion Medium. For most of the iPSC lines, the neural induction protocol generated cells with characteristic NSC-like morphology (Fig. 4B), although two clones (32517-A and 32643-C) consistently detached during induction and could not be converted to NSCs. At the initial plating (immediately after seven days of induction), NSC cultures occasionally contained clusters of cells resembling small iPSC colonies, potentially suggesting residual pluripotent cells. After 1-2 passages in culture, these cells were no longer apparent, and immunostaining with an antibody for Oct3/4 detected no positive cells (data not shown). By passage 3 post-induction, XDP and control cultures typically consisted predominantly of cells immunopositive for nestin, musashi and Sox1 (Fig. 4C-E). For a quantitative assessment of neural conversion, RNA from all NSC lines, as well as the parent iPSC clones, was assayed by qRT-PCR using a panel of primers interrogating 22 genes known to be expressed in neural stem cells (Fig. 4F). Expression of all neural marker genes was low and/or non-detectable in iPSC clones and increased in NSCs with only few exceptions. Control iPSC clone 33362-D exhibited high expression of *APBB1* and *CDK5RAP2*, unlike the other iPSC clones, whereas XDP NSC line 33363-C showed low expression of *DLL* and *EGF*, in contrast to the other NSC lines. Yet despite some variability in expression level of individual genes among the cell lines, assessment of the full panel of markers revealed no patterns to indicate any genotypic differences in neural conversion between XDP and control cells. Furthermore, no NSC clone differed consistently from all other lines across the entire panel that might suggest aberrant differentiation. Based on these data, the final set of NSCs used for analysis of *TAF1* transcript expression consisted of 7 XDP clones derived from five patients versus six control clones from three individuals.

### Analysis of *TAF1* and MTS transcripts in NSCs

**Fig. 5.**
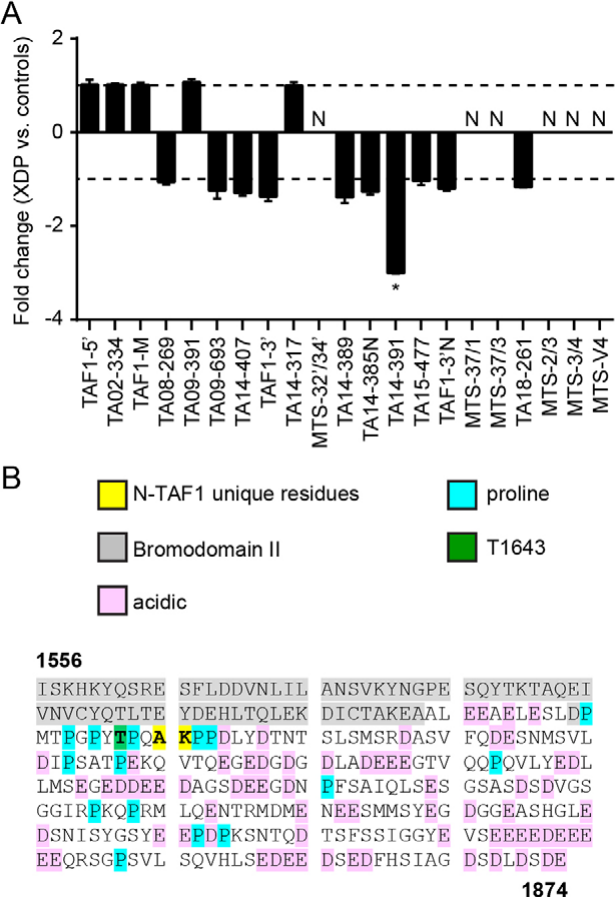
***TAF1* and MTS transcript expression levels in NSCs.** Quantitative expression analysis of *TAF1* transcript fragments in XDP (*n*=7) vs control (*n*=6) NSC clones based on comparative Ct method with targets normalized to the geometric mean of housekeeping genes *HPRT1* and *TFRC*. (A) N-TAF1, amplified by primer set TA14-391, was detected in NSCs at levels approximately threefold lower in XDP vs control cells. Data represent mean fold changes±standard errors, analyzed by Student's *t*-test. *P<0.05; N, not detected. (B) Amino acids 1556-1874 of N-TAF1 highlighted to indicate unique alanine (A) and lysine (K) residues relative to: C-terminal segment of second bromodomain motif (gray); acidic glutamate (E) and aspartate (D) residues (pink); prolines (P; blue); and a threonine at position 1643 (T1643; green) that is a potential phosphorylation site.

We used the panel of primers interrogating *TAF1* and MTS-derived mRNAs to compare transcriptional patterns in XDP and control NSCs. RNA was isolated from all NSC lines at passage 4 post-induction. As in the fibroblasts, the NSC lines did not seem to express any of the MTS-derived RNAs (Fig. 5A). Unlike in fibroblasts, the N-TAF1 variant was expressed in NSCs at levels that were on average ∼threefold lower in XDP versus control clones (*P*<0.05; Fig. 5A). Although multiple TAF1 fragments seemed to be differentially expressed in XDP versus control fibroblasts (Fig. 1B), N-TAF1 was the only transcript in this panel that distinguished XDP from control NSCs. N-TAF1 contains an alternative exon, 34′ (Fig. 1C), consisting of only six nucleotides that encode two additional residues, an alanine and a lysine, which become positions 1646-1647, respectively, of a 1847-aa protein (Fig. 5B) (Makino et al., 2007). The insertion is C-terminal to two tandem bromodomains, which mediate histone interactions (Jacobson et al., 2000), and immediately adjacent to a threonine, T1643, which is a potential phosphorylation site (Hornbeck et al., 2012). These three residues, T1643, A1646 and K1647, fall within a short cluster of prolines that could potentially form a polyproline helix, although it is difficult to accurately predict such motifs *in silico*. The other notable feature of this segment is the preponderance of aspartate and glutamate residues that seem to make up an acidic tail. Aside from these limited observations, the protein sequence itself does not reveal potential function(s) of the N-TAF1-unique residues, and because the insertion falls within an intrinsically disordered region of the protein, structural analyses of this segment have not been possible (Jacobson et al., 2000).

## DISCUSSION

XDP was first described by Lee et al. (1976) as torsion dystonia of Panay, with 19 of 23 cases coming from the province of Capiz in the Philippines. Since that pioneering report, the natural history of XDP has been documented in detail, facilitated by the formation of an XDP Study Group and an XDP Registry (Lee et al., 2011). As of January 2010, this registry consisted of 505 cases corresponding to an approximate prevalence of 5.74 cases per 100,000 individuals in Panay (1:4000 men specifically in Capiz), or 0.31:100,000 within the entire Philippine population (Lee et al., 2011). The clinical profiles of these cases, as well as ones from other centers, consist of an adult-onset movement disorder that typically begins as focal dystonia that generalizes over time and is combined with or replaced by parkinsonian features (Lee et al., 1991, 2002, 2011; Evidente et al., 2002a). In a small percentage of documented cases, parkinsonian symptoms appear as the initial clinical findings (Lee et al., 2002; Evidente et al., 2002b), but in most individuals characterized to date, disease onset involved focal dystonia of either the lower extremities, craniofacial musculature (e.g. oromandibular dystonia, blepharospasm) or upper extremities (Lee et al., 2002). The vast majority of affected individuals are males, but there are reports of symptomatic females resulting from homozygosity of the disease allele, skewed X-inactivation or Turner's syndrome (Lee et al., 2001; Evidente et al., 2004; Westenberger et al., 2013; Domingo et al., 2014, 2015). Among affected individuals, disease progression is variable, but the most frequent clinical picture involves spread of dystonic symptoms during the first decade following diagnosis, combined with the emergence of parkinsonian symptoms that predominate by 15 years after the initial onset (Lee et al., 2002).

The XDP subjects investigated here appeared consistent with previous reports in terms of clinical presentations and positive responses to pallidal DBS, which has been documented by multiple groups (Evidente et al., 2007; Wadia et al., 2010; Oyama et al., 2010; Aguilar et al., 2011). Another consistent finding was the combined presence of multiple haplotype markers. The most recent effort to narrow the XDP genomic region involved genotyping of 163 males with clinical diagnoses of XDP and 473 Filipino controls, of which 452 had ancestry specifically to Panay (Domingo et al., 2015). The seven known disease-related variants (five DSCs, a 48-bp deletion and the SVA; Nolte et al., 2003; Makino et al., 2007) were found in all but five of the affected individuals; the latter group, thought to be phenocopies, were negative for all markers, similar to the control subjects (Domingo et al., 2015). In this study, we genotyped six of the seven sequence variants and observed a similar ‘all or none’ distribution in affected versus unaffected family members. This inability to rule out any of the seven genomic variants has complicated efforts to understand XDP pathogenesis. That these sequence variants cluster around *TAF1* and the intergenic MTS has focused most hypothesized disease models on their respective gene products, but crucial questions remain unanswered. With respect to the MTS, it is noteworthy that it contains the only XDP sequence variant, DSC3, which alters a potential coding exon (Nolte et al., 2003; Herzfeld et al., 2007). Furthermore, ectopic expression of wild-type or mutated (DSC3) MTS cDNAs in control cell lines resulted in different transcriptional profiles, suggesting that the DSC3 substitution could have functional effects (Herzfeld et al., 2013). However, very little is known about the normal distribution of endogenous MTS transcripts, the type(s) of protein(s) that might be generated from these mRNAs, and what role, if any, they might play within neural structures thought to be affected in XDP.

TAF1, by contrast, has been extensively characterized as part of the general transcriptional machinery. The human gene was cloned in the effort to define co-activators that associate with the TATA-binding protein (TBP) within the general TFIID complex (Ruppert et al., 1993). It proved identical to a previously discovered cell cycle-related gene, *CCG1*, in which a temperature-sensitive point mutation prevents G1-S progression in the hamster cell line, tsBN462 (Sekiguchi et al., 1988, 1991). TFIID is involved in transcription initiation by RNA polymerase II (Grünberg and Hahn, 2013; Kandiah et al., 2014). It is a multi-subunit complex composed of TBP and at least 13 TAFs that collectively bind the TATA element upstream of the transcription start site in many gene promoters (Papai et al., 2011; Thomas and Chiang, 2006). The major isoform of TAF1 is a large, 250 kDa protein containing an N-terminal domain that binds TBP in competition with other TFIID components (Anandapadamanaban et al., 2013). TAF1 seems to be a negative regulator of TBP, inhibiting its DNA-binding activity and removing TFIID from the TATA element, which might be crucial in determining the balance between transcription initiation and termination (Thomas and Chiang, 2006). Structural analyses further suggest that TAF1 might have intrinsic DNA-binding activity itself. The conserved central core of TAF1 contains a winged helix (WH) domain that directly binds DNA and might mediate the effects of TAF1 on expression of various cell cycle genes (Wang et al., 2014). Thus, it is possible that TAF1 might act as a gene-specific transcription factor independent of its major role in modulating TBP within TFIID.

The SVA insertion within intron 32 of *TAF1* is an intriguing candidate as the XDP genetic lesion, given the growing recognition of how mobile DNA elements might cause human disease. SVAs represent one of the many types of retrotransposon insertions, including LINEs (L1s), Alus, SINEs and human endogenous retrovirus (HERV) sequences, that comprise over 40% of the human genome (Hancks and Kazazian, 2012). Although evolutionarily millions of years old, these elements retain some potential to insert at new sites and cause disease. There are 2676 SVA elements comprising 0.13% of the human genome, with a new insertion in one out of every 916 births (Savage et al., 2013; Cordaux and Batzer, 2009; Hancks and Kazazian, 2010). SVA retrotransposition depends on endonuclease and reverse transcriptase activities derived from L1 elements, a process known as target site-primed reverse transcription. SVA inserts vary from 700 bp to 4 kb, with structures typically consisting of: (1) a 5′ to 3′ hexameric CCCTC repeat; (2) two anti-sense Alus; (3) a variable number of tandem GC-rich repeats; (4) shared sequence identity with the *env* gene and the right long terminal repeat of HERV-K10; and (4) a polyA signal sequence. The repetitive nature of these sequences throughout the genome renders them difficult to distinguish individually. There are over 12 human diseases, including cancer, known to be caused by SVA insertions, most of which are inserted in the sense orientation. Documented effects of such insertions include: (1) deletions of endogenous gene sequences, as in *NF1* (Vogt et al., 2014) and the promoter of *FUS* (Savage et al., 2013); (2) insertions associated with cancer (Szpakowski et al., 2009); (3) altered splicing, including exon skipping (*SPTA1*, *BTK*); (4) exonization of SVA into transcripts, which then become out-of-frame and dysfunctional (*FKTN*, *ARH*, *LDLRAP1*, *PNPLA2*, *PMS2*; Kwon et al., 2013; Hancks et al., 2009); and (5) transcription of SVA sequences driven by internal retrotransposon elements (Hancks and Kazazian, 2010).

Our transcriptional profiling detected significant differences in the expression of multiple *TAF1* transcript fragments in XDP versus control fibroblasts, whereas only N-TAF1 seemed to be differentially expressed in the iPSC-derived NSCs. A previous study similarly assayed levels of different *TAF1* transcripts in lymphocytes from a single XDP individual versus 60 control subjects (Deng et al., 2008) but detected no significant changes in expression, which might reflect the limitations of analyzing a single proband and/or patterns specific to lymphocytes. Tissue-specific alternative splicing of *TAF1* has been previously reported, which might, in some cells, be regulated at least in part by ataxia-telangiectasia-mutated (ATM) and ATM-RAD3-related (ATR) pathways (Katzenberger et al., 2006; Marengo and Wassarman, 2008). However, the signals governing neural-specific expression of N-TAF1 are not known. The decreased expression of N-TAF1 detected here seems consistent with the decrease reported in XDP caudate (Makino et al., 2007), indicating that this phenotype in XDP brain tissue is reproduced in the iPSC-derived NSCs. The neural cells differentiated here generally resembled early stage progenitors capable of generating both neuronal and glial cell types, which might suggest that aberrant N-TAF1 expression occurs at early stages that precede the development of neurodegeneration in XDP individuals. In addition to a selective loss of striatal medium spiny neurons (Goto et al., 2005), XDP brains also exhibit decreased numbers of proliferating cell nuclear antigen (PCNA)-positive neural progenitors within the subventricular zone, compared with control tissue (Goto et al., 2013). Thus, the recent neuropathology data and the present findings both seem to suggest that XDP-related functional defects might develop not only in mature neurons but also in neural precursor cells.

A curious feature of N-TAF1 is that it differs from the major isoform of TAF1 by only two amino acids. That these residues fall within an intrinsically disordered region of the protein prevents any structural modeling that might reveal a functional significance for this insertion. The surrounding cluster of prolines could indicate a polyproline helix, which can be a recognition motif involved in diverse cellular functions, including transcription (Adzhubei et al., 2013). If this segment forms a local helical structure, then the insertion of alanine and lysine would alter the configuration by changing the spacing of the prolines. Such a rearrangement might affect interactions with other proteins at this site. Nevertheless, such possibilities remain speculative at this point and will require empirical testing.

If the pathogenic defect in XDP is in fact reduced expression of several TAF1 isoforms, it might seem surprising that the medical phenotype is exclusively neurologic, given the role of different TAF1 isoforms in fundamental aspects of transcription in all cells. Yet such a scenario would not be unique, as sequence variations in TBP, the canonical binding partner for TAF1 and arguably the most crucial component of the TFIID complex, are also associated with neurodegeneration. Expanded trinucleotide repeat sequences in TBP have been shown to cause spinocerebellar ataxia-17 (SCA17) and to phenocopy Huntington's disease (Nakamura et al., 2001; Fujigasaki et al., 2001; Stevanin et al., 2003; Hsu et al., 2014). This observation reinforces the hypothesis that at least some perturbations within TFIID have consequences specific to the central nervous system (CNS). Moreover, XDP might not be the only link between TAF1 and neurologic disease. Recent sequencing efforts have linked different coding mutations in TAF1 to X-linked intellectual disability (Hu et al., 2016). Thus, it is possible that XDP could be part of a spectrum of TAF1-related disorders in which different mutations that affect the protein to different extents induce CNS disorders of varying degrees of severity.

It should be noted that the lack of MTS expression in the cells profiled here does not in itself exclude the potential importance of these exons and/or DSC3 to XDP. It is still possible that these transcripts might be expressed at later neurodevelopmental stages and/or in specific neuronal subtypes that are involved in the disease process. Further studies will therefore be required to probe the possible contributions of these transcripts as well as the specific cellular pathways that might be affected by them and/or by decreased N-TAF1 expression. In that regard, the development of XDP patient-specific iPSC lines that can be differentiated toward specific neuronal cell fates in culture might be powerful tools that enable such investigations.

## MATERIALS AND METHODS

### XDP and control subjects

Subjects recruited for this study included: (1) individuals with a confirmed diagnosis of XDP based on prior genetic testing; (2) individuals suspected of XDP based on clinical history and Filipino ancestry; and (3) unaffected family members. The study was approved by the institutional review board at Massachusetts General Hospital, and all participants gave written informed consent. Clinical evaluation included comprehensive neurological exams with recorded scores for standard scales: Burke–Fahn–Marsden, Tsui–Torticollis, Toronto Western Spasmodic Torticollis Rating and Voice Disability Index (Albanese et al., 2013b).

### XDP and control fibroblasts

For fibroblast derivation, skin biopsies were performed by standard procedures (Wray et al., 2012). Tissue explants were transferred to culture dishes containing fibroblast growth medium [Dulbecco's Modified Eagle Medium (DMEM) with 20% fetal bovine serum (FBS) and 1% penicillin/streptomycin] and placed under sterile coverslips to promote cell attachment. Cultures were maintained in a humidified incubator at 37°C and 5% CO_2_ with medium exchanges every 3-4 days as fibroblasts migrated from the explant. Cultures were expanded by trypsinization, collecting cells by centrifugation, and resuspending in growth medium. All tissue culture media and supplements were obtained from Life Technologies (Grand Island, NY, USA) except where otherwise noted. All cell lines were confirmed to be free of contamination, including mycoplasma, prior to experimental use.

### Genotyping

Genomic DNA (gDNA) was isolated from cells using DNeasy Blood and Tissue kit (Qiagen, Valencia, CA, USA), as recommended. Genotyping was performed by PCR amplification of regions bearing six previously reported haplotype markers: five disease-specific single nucleotide changes (DSC1, -2,-3,-10,-12; Nolte et al., 2003) and the SVA in intron 32 of *TAF1* (Makino et al., 2007). Primers used for PCR amplification of DSCs (Table S1) included previously published sequences (Nolte et al., 2003), as well as novel primers generated using NCBI Primer-BLAST software. PCR amplicons were resolved by gel electrophoresis, purified using QIAquick Gel Extraction kit (Qiagen), and sequenced to determine the presence or absence of XDP-specific nucleotide substitutions. To detect the SVA, long-range PCR was performed using previously described primers (Kawarai et al., 2013) and Takara's PrimeSTAR GXL DNA polymerase (Clontech, Mountain View, CA, USA) with modified amplification conditions (Table S1). PCR amplicons were resolved by gel electrophoresis to identify either a 3229 bp product (XDP; containing the ∼2.6 kb SVA) or a 599 bp product (control; no SVA).

### iPSC reprogramming and confirmation of pluripotency

To generate iPSCs, XDP and control fibroblasts were seeded into six-well plates at an approximate density of 1×10^5^ cells/well in fibroblast growth medium. Reprogramming was performed using the Cytotune-iPS Reprogramming kit (Life Technologies) consisting of non-integrating Sendai viruses encoding the classical Yamanaka factors, Oct3/4, Sox2, Klf4 and c-Myc (Takahashi and Yamanaka, 2006). When fibroblasts reached ∼80% confluence, they were transduced with four viruses at approximate multiplicity of infection (MOI) of 3. Cells were maintained for one week with medium exchanges every other day before collecting and re-seeding into gelatin-coated dishes containing feeder layers of irradiated CF1 mouse embryonic fibroblasts (MEFs; GlobalStem, Gaithersburg, MD, USA). Transduced cells on feeder layers were then switched to human embryonic stem cell (hESC) medium consisting of DMEM:F12 supplemented with 7 μl/l 2-mercaptoethanol (2-ME; Sigma, St Louis, MO, USA), 20% Knockout Serum Replacement (KOSR), 2× L-Glutamine, 1× Minimum Eagle's Medium-Non-Essential Amino Acids (MEM-NEAA) and 10 ng/ml basic fibroblast growth factor (bFGF). Colonies were initially picked manually for transfer to fresh feeder layers. Subsequent expansion was achieved by digestion in collagenase IV, followed by scraping and centrifugation to collect cells. Pellets were triturated gently in fresh hESC medium to generate medium-small clumps that were seeded onto fresh feeder layers.

To confirm reprogramming and assess pluripotency, RNA was isolated from colonies using RNeasy reagents and spin columns (Qiagen) with on-column DNase digestion and then reverse transcribed using High Capacity cDNA Reverse Transcription kit (Life Technologies), all as recommended. Expression of endogenous *Dnmt3B*, *hTERT*, *Nanog*, *Rex1*, *Oct4* and *Sox2* genes, as well as housekeeping gene *ACTB*, was quantified by qRT-PCR in each iPSC clone as well as the corresponding parent fibroblast line. To generate differentiated EBs bearing cells of all three germ layers, colonies were collected from Matrigel-coated plates and seeded onto ultra-low attachment plates (Corning, Tewksbury, MA, USA) in hESC medium without bFGF and maintained for 1 week. Suspension culture EBs were then re-plated on 0.1% gelatin-coated plates in DMEM/10% FBS/1% penicillin/streptomycin and maintained for an additional week of adherent culture at which point RNA was isolated and reverse transcribed. The resulting cDNA samples, along with cDNA prepared from the undifferentiated, parent iPSC clones, were used for quantitative analysis of pluripotency using the TaqMan hPSC Scorecard Assay as recommended (Thermo Fisher Scientific). The assay panel consisted of 94 marker genes to evaluate trilineage potential. Quantitative analysis of gene expression data was performed using hPSC Scorecard analysis software (Thermo Fisher Scientific). All gene expression data were visualized as heatmaps generated using R software (www.R-project.org).

To further confirm pluripotency *in vivo*, ∼1×10^6^ cells of selected clones were resuspended in a mixture of phosphate-buffered saline (PBS) and Matrigel, then implanted transcutaneously at multiple sites in Fox Chase SCID mice (Charles River, Wilmington, MA, USA). Mice were euthanized after 6-8 weeks by CO_2_ inhalation. Tumors were collected, embedded in paraffin, sectioned, and stained with H&E to identify cellular morphologies characteristic of all three germ layers. Cell lines were confirmed to be karyotypically normal by Cell Line Genetics, Inc. (Madison, WI, USA). All animal experiments were performed according to procedures approved by the Institutional Animal Care and Use Committee (IACUC) of Harvard University.

For routine propagation of XDP and control iPSC lines, cells were adapted for growth under feeder-free conditions on Geltrex-coated tissue culture plates in mTESR-1 medium (Stemcell Technologies, Vancouver, BC, Canada). Passaging of colonies on Geltrex was performed by initial digestion in Accutase, followed by scraping, as described above. Cell lines were routinely tested for contamination, including mycoplasma, and confirmed to be negative.

### Neural differentiation

XDP and control iPSC colonies on Geltrex were dissociated in Accutase to single cells that were then seeded into fresh coated plates at an approximate density of 2-3×10^5^ cells/well in mTESR-1 medium containing 10 μM ROCK inhibitor, Y-27632. Medium was changed the next day to remove ROCK inhibitor. When cells reached ∼25% confluence, they were placed in PSC Neural Induction Medium for seven days with medium exchanges every other day. On day eight, cells were detached using Accutase, collected by centrifugation, and seeded onto fresh coated plates in neural expansion medium consisting of Neurobasal Medium: DMEM/F12 (1:1) supplemented with 2% PSC Neural Induction Supplement and 5 μM Y-27632. After 24 h, medium was exchanged again to remove ROCK inhibitor. The resulting NSCs were maintained on Geltrex-coated plates in neural expansion medium for up to 4 or 5 passages in culture. NSCs were confirmed to be immune-positive for neural markers, nestin, musashi and Sox1, but negative for pluripotent marker, Oct3/4, by antibody staining. For a quantitative assessment of neural conversion, RNA from differentiated NSCs and the parent iPSC clones was reverse transcribed and subjected to qPCR using a panel of primers interrogating 22 marker genes for neural stem cells (Qiagen).

The human neuroblastoma cell line, SH-SY5Y (ATCC; Manassas, VA USA), was also used as a control to confirm neural specificity of N-TAF1. SH-SY5Y cells were cultured in DMEM containing 10% FBS and 1× penicillin/streptomycin and passaged using trypsin. Differentiation was induced by addition of 10 μM ATRA (Sigma) to the culture medium for 72 h. Differentiation was confirmed based on characteristic changes in morphology, consisting of long branched neurites.

### Immunofluorescence

Immunostaining was performed to confirm expression of pluripotent marker proteins in iPSC lines and neural marker proteins in NSCs. Primary antibodies and dilutions used in this study consisted of the following: rabbit anti-Oct3/4 (Abcam, ab19857; 1:100), rabbit anti-NANOG (Abcam ab21624; 1:50), Rat anti-SSEA-3 (Millipore, MAB4303; 1:200), mouse anti-SSEA-4 (Millipore, MAB4304; 1:200), mouse anti-Tra-1-60 (EMD Millipore MAB4360; 1:200), mouse anti-nestin (Abcam, ab21628; 1:200), rabbit anti-musashi (Abcam, ab21628; 1:100) and Sox1 (Abcam, ab87775; 1:250). Staining was detected using the following additional reagents (all from Life Technologies): goat anti-rabbit Alexa Fluor 488 (A-11008) and Alexa Fluor 594 (A-11012), goat anti-rat Alexa Fluor 594 (A-11007), goat anti-mouse Alexa Fluor 488 (A-11001) and Alexa Fluor 594 (A-11032), all at a final dilution of 1:1000. In some experiments, wheat germ agglutinin-Alexa Fluor 488 (W11261; 1:200) and Topro-3-iodide (T3605; 1:200) were included as counterstains to visualize plasma membranes and nuclei, respectively. For immunostaining, cells seeded into chamber slides were fixed in 4% paraformaldehyde for 20 min at room temperature, washed 3× in phosphate buffered saline (PBS), permeabilized in 0.1% Nonidet P-40 for 20 min, rinsed 3× again in PBS, and then blocked in normal goat serum (10% in PBS) for 1 h. Cells were incubated in primary antibodies at the specified dilution overnight at 4°C. The next day, slides were washed 3× in PBS, incubated in secondary antibodies for 45 min, and then counterstained (where indicated) for 15 min.

### RT-PCR for *TAF1* and MTS transcripts

To quantify expression of transcripts derived from exons 1-38 of *TAF1*, as well as the unconventional exons of the MTS (Nolte et al., 2003), RT-PCR was performed using a panel of previously described primers (Makino et al., 2007). RNA was extracted from XDP and control fibroblasts and iPSC-derived NSCs using Qiazol (Qiagen), then mixed with 1/5th volume of chloroform with brief centrifugation to allow phase separation. The aqueous phase was further processed using miRNeasy spin columns (Qiagen) with on-column DNase digestion, as recommended. The resulting RNA samples were quantified by Nanodrop, and equivalent amounts were reverse transcribed using RT^2^ First Strand kit (Qiagen), which includes an additional DNase digestion to remove any residual contaminating gDNA. PCR was carried out with 87.6 ng cDNA in 20 µl reactions using TaqMan Gene Expression Master Mix (Life Technologies) on a StepOne Plus Real-Time PCR system (Life Technologies). Amplification conditions consisted of holding stage of 50°C for 2 min and 95°C for 10 min followed by 40 cycles of 95°C for 15 s and 60°C for 1 min. Data were analyzed with RT² Profiler PCR Array Data Analysis software v3.5 (Qiagen) using the ΔΔCt method and normalized to housekeeping genes. Multiple candidate housekeeping genes were initially screened to identify transcripts displaying stable expression across all cell lines at levels roughly equivalent to the *TAF1*-related transcripts. Of the housekeeping genes tested, *HPRT*, *TFRC* and *GAPDH* gave the most consistent levels and were thus used for normalization. Within each experiment, Ct values for each target were normalized to the geometric mean of the housekeeping genes. For statistical analysis, RT² Profiler software calculated *P* values based on Student's *t*-tests of replicate 2^−ΔCt^ values for each target transcript in XDP versus control cells. Raw Ct values for all gene expression data are listed in Tables S2-S5.

For additional validation of the specificity of primers targeting the brain-enriched isoform, N-TAF1 (Makino et al., 2007), RT-PCR was also performed on RNA extracted from (1) human SH-SY5Y neuroblastoma cells, with and without ATRA-mediated differentiation; and (2) human brain (Biochain; Newark, CA, USA).

To probe for potential exonization of the SVA, cDNA samples from XDP cells were amplified with two sets of primers flanking the SVA insertion site (Table 1) in all four combinations. Because incorporation of aberrant SVA sequences into *TAF1*-derived transcripts could potentially target RNAs for NMD, cells were cultured for 6 h in growth medium containing 50 μM of a specific NMD inhibitor, ethyl 2-{[(6,7-dimethyl-3-oxo-1,2,3,4-tetrahydro-2-quinoxalinyl)acetyl]amino}-4,5-dimethyl-3-thiophenecar-boxylate (NMDI14; Martin et al., 2014), prior to RNA isolation. PCR products were evaluated based on melting temperature profiles, apparent size in gel electrophoresis, and Sanger sequencing.

Additional qRT-PCR was performed to amplify three genes also contained with the XDP chromosomal region (*CXCR3*, *ARC*, *OGT*) using commercially available Taqman primers (Life Technologies).

## References

[DMM022590C1] Adzhubei A. A., Sternberg M. J. E. and Makarov A. A. (2013). Polyproline-II helix in proteins: structure and function. *J. Mol. Biol.* 425, 2100-2132. 10.1016/j.jmb.2013.03.018 23507311

[DMM022590C2] Aguilar J. A., Vesagas T. S., Jamora R. D., Teleg R. A., Ledesma L., Rosales R. L., Fernandez H. H. and Lee L. V. (2011). The promise of deep brain stimulation in X-linked dystonia parkinsonism. *Int. J. Neurosci.* 121, 57-63. 10.3109/00207454.2010.541573 21244299

[DMM022590C3] Albanese A., Bhatia K., Bressman S. B., DeLong M. R., Fahn S., Fung V. S. C., Hallett M., Jankovic J., Jinnah H. A., Klein C. et al. (2013a). Phenomenology and classification of dystonia: a consensus update. *Mov. Disord.* 28, 863-873. 10.1002/mds.25475 23649720 PMC3729880

[DMM022590C4] Albanese A., Sorbo F. D., Comella C., Jinnah H. A., Mink J. W., Post B., Vidailhet M., Volkmann J., Warner T. T., Leentjens A. F. et al. (2013b). Dystonia rating scales: critique and recommendations. *Mov. Disord.* 28, 873-874. 10.1002/mds.25579 PMC4207366 23893443

[DMM022590C5] Anandapadamanaban M., Andresen C., Helander S., Ohyama Y., Siponen M. I., Lundström P., Kokubo T., Ikura M., Moche M. and Sunnerhagen M. (2013). High-resolution structure of TBP with TAF1 reveals anchoring patterns in transcriptional regulation. *Nat. Struct. Mol. Biol.* 20, 1008-1014. 10.1038/nsmb.2611 23851461 PMC4972576

[DMM022590C6] Bock C., Kiskinis E., Verstappen G., Gu H., Boulting G., Smith Z. D., Ziller M., Croft G. F., Amoroso M. W., Oakley D. H. et al. (2011). Reference maps of human ES and iPS cell variation enable high-throughput characterization of pluripotent cell lines. *Cell* 144, 439-452. 10.1016/j.cell.2010.12.032 21295703 PMC3063454

[DMM022590C7] Camargos S., Lees A. J., Singleton A. and Cardoso F. (2012). DYT16: the original cases. *J. Neurol. Neurosurg. Psychiatry* 83, 1012-1014. 10.1136/jnnp-2012-302841 22842711 PMC6376866

[DMM022590C8] Cordaux R. and Batzer M. A. (2009). The impact of retrotransposons on human genome evolution. *Nat. Rev. Genet.* 10, 691-703. 10.1038/nrg2640 19763152 PMC2884099

[DMM022590C9] Deng H., Le W.-D. and Jankovic J. (2008). Genetic study of an American family with DYT3 dystonia (lubag). *Neurosci. Lett.* 448, 180-183. 10.1016/j.neulet.2008.10.049 18952144

[DMM022590C10] Domingo A., Lee L. V., Brüggemann N., Freimann K., Kaiser F. J., Jamora R. D. G., Rosales R. L., Klein C. and Westenberger A. (2014). Woman with x-linked recessive dystonia-parkinsonism: clue to the epidemiology of parkinsonism in Filipino women? *JAMA Neurol.* 71, 1177-1180. 10.1001/jamaneurol.2014.56 25004170

[DMM022590C11] Domingo A., Westenberger A., Lee L. V., Brænne I., Liu T., Vater I., Rosales R., Jamora R. D., Pasco P. M., Cutiongco-Dela Paz E. M. et al. (2015). New insights into the genetics of X-linked dystonia-parkinsonism (XDP, DYT3). *Eur. J. Hum. Genet.* 23, 1334-1340. 10.1038/ejhg.2014.292 25604858 PMC4592086

[DMM022590C12] Evidente V. G. H., Advincula J., Esteban R., Pasco P., Alfon J. A., Natividad F. F., Cuanang J., Luis A. S., Gwinn-Hardy K., Hardy J. et al. (2002a). Phenomenology of “Lubag” or X-linked dystonia-parkinsonism. *Mov. Disord.* 17, 1271-1277. 10.1002/mds.10271 12465067

[DMM022590C13] Evidente V. G., Gwinn-Hardy K., Hardy J., Hernandez D. and Singleton A. (2002b). X-linked dystonia (“Lubag)” presenting predominantly with parkinsonism: a more benign phenotype? *Mov. Disord.* 17, 200-202. 10.1002/mds.1263 11835466

[DMM022590C14] Evidente V. G. H., Nolte D., Niemann S., Advincula J., Mayo M. C., Natividad F. F. and Müller U. (2004). Phenotypic and molecular analyses of X-linked dystonia-parkinsonism (“Lubag)” in women. *Arch. Neurol.* 61, 1956-1959. 10.1001/archneur.61.12.1956 15596620

[DMM022590C15] Evidente V. G. H., Lyons M. K., Wheeler M., Hillman R., Helepolelei L., Beynen F., Nolte D., Müller U. and Starr P. A. (2007). First case of X-linked dystonia-parkinsonism (“Lubag”) to demonstrate a response to bilateral pallidal stimulation. *Mov. Disord.* 22, 1790-1793. 10.1002/mds.21420 17579361

[DMM022590C16] Fujigasaki H., Martin J.-J., De Deyn P. P., Camuzat A., Deffond D., Stevanin G., Dermaut B., Van Broeckhoven C., Dürr A. and Brice A. (2001). CAG repeat expansion in the TATA box-binding protein gene causes autosomal dominant cerebellar ataxia. *Brain* 124, 1939-1947. 10.1093/brain/124.10.1939 11571212

[DMM022590C17] Geyer H. L. and Bressman S. B. (2011). Rapid-onset dystonia-parkinsonism. *Handb. Clin. Neurol.* 100, 559-562. 10.1016/B978-0-444-52014-2.00040-9 21496607

[DMM022590C18] Goto S., Lee L. V., Munoz E. L., Tooyama I., Tamiya G., Makino S., Ando S., Dantes M. B., Yamada K., Matsumoto S. et al. (2005). Functional anatomy of the basal ganglia in X-linked recessive dystonia-parkinsonism. *Ann. Neurol.* 58, 7-17. 10.1002/ana.20513 15912496

[DMM022590C19] Goto S., Kawarai T., Morigaki R., Okita S., Koizumi H., Nagahiro S., Munoz E. L., Lee L. V. and Kaji R. (2013). Defects in the striatal neuropeptide Y system in X-linked dystonia-parkinsonism. *Brain* 136, 1555-1567. 10.1093/brain/awt084 23599389

[DMM022590C20] Graeber M. B. and Müller U. (1992). The X-linked dystonia-parkinsonism syndrome (XDP): clinical and molecular genetic analysis. *Brain Pathol.* 2, 287-295. 10.1111/j.1750-3639.1992.tb00706.x 1364136

[DMM022590C21] Grünberg S. and Hahn S. (2013). Structural insights into transcription initiation by RNA polymerase II. *Trends Biochem.* 38, 603-611. 10.1016/j.tibs.2013.09.002 PMC3843768 24120742

[DMM022590C22] Haberhausen G., Schmitt I., Köhler A., Peters U., Rider S., Chelly J., Terwilliger J. D., Monaco A. P. and Müller U. (1995). Assignment of the dystonia-parkinsonism syndrome locus, DYT3, to a small region within a 1.8-Mb YAC contig of Xq13.1. *Am. J. Hum. Genet.* 57, 644-650. 7668293 PMC1801270

[DMM022590C23] Hancks D. C. and Kazazian H. H. (2010). SVA retrotransposons: evolution and genetic instability. *Semin. Cancer Biol.* 20, 234-245. 10.1016/j.semcancer.2010.04.001 20416380 PMC2945828

[DMM022590C24] Hancks D. C. and Kazazian H. H. (2012). Active human retrotransposons: variation and disease. *Curr. Opin. Genet. Dev.* 22, 191-203. 10.1016/j.gde.2012.02.006 22406018 PMC3376660

[DMM022590C25] Hancks D. C., Ewing A. D., Chen J. E., Tokunaga K. and Kazazian H. H. (2009). Exon-trapping mediated by the human retrotransposon SVA. *Genome Res.* 19, 1983-1991. 10.1101/gr.093153.109 19635844 PMC2775590

[DMM022590C26] Hanna J. H., Saha K. and Jaenisch R. (2010). Pluripotency and cellular reprogramming: facts, hypotheses, unresolved issues. *Cell* 143, 508-525. 10.1016/j.cell.2010.10.008 21074044 PMC3032267

[DMM022590C27] Herzfeld T., Nolte D. and Müller U. (2007). Structural and functional analysis of the human TAF1/DYT3 multiple transcript system. *Mamm. Genome* 18, 787-795. 10.1007/s00335-007-9063-z 17952504

[DMM022590C28] Herzfeld T., Nolte D., Grznarova M., Hofmann A., Schultze J. L. and Müller U. (2013). X-linked dystonia parkinsonism syndrome (XDP, lubag): disease-specific sequence change DSC3 in TAF1/DYT3 affects genes in vesicular transport and dopamine metabolism. *Hum. Mol. Genet.* 22, 941-951. 10.1093/hmg/dds499 23184149

[DMM022590C29] Hornbeck P. V., Kornhauser J. M., Tkachev S., Zhang B., Skrzypek E., Murray B., Latham V. and Sullivan M. (2012). PhosphoSitePlus: a comprehensive resource for investigating the structure and function of experimentally determined post-translational modifications in man and mouse. *Nucleic Acids Res.* 40, D261-D270. 10.1093/nar/gkr1122 22135298 PMC3245126

[DMM022590C30] Hsu T.-C., Wang C.-K., Yang C.-Y., Lee L.-C., Hsieh-Li H.-M., Ro L.-S., Chen C.-M., Lee-Chen G.-J. and Su M.-T. (2014). Deactivation of TBP contributes to SCA17 pathogenesis. *Hum. Mol. Genet.* 23, 6878-6893. 10.1093/hmg/ddu410 25104854

[DMM022590C31] Hu H., Haas S. A., Chelly J., Van Esch H., Raynaud M., de Brouwer A. P., Weinert S., Froyen G., Frints S. G., Laumonnier F. et al. (2016). X-exome sequencing of 405 unresolved families identifies seven novel intellectual disability genes. *Mol. Psychiatry* 21, 133-148. 10.1038/mp.2014.193 25644381 PMC5414091

[DMM022590C32] Jacobson R. H., Ladurner A. G., King D. S. and Tjian R. (2000). Structure and function of a human TAFII250 double bromodomain module. *Science* 288, 1422-1425. 10.1126/science.288.5470.1422 10827952

[DMM022590C33] Jambaldorj J., Makino S., Munkhbat B. and Tamiya G. (2012). Sustained expression of a neuron-specific isoform of the Taf1 gene in development stages and aging in mice. *Biochem. Biophys. Res. Commun.* 425, 273-277. 10.1016/j.bbrc.2012.07.081 22842574

[DMM022590C34] Kandiah E., Trowitzsch S., Gupta K., Haffke M. and Berger I. (2014). More pieces to the puzzle: recent structural insights into class II transcription initiation. *Curr. Opin. Struct. Biol.* 24, 91-97. 10.1016/j.sbi.2013.12.005 24440461

[DMM022590C35] Katzenberger R. J., Marengo M. S. and Wassarman D. A. (2006). ATM and ATR pathways signal alternative splicing of Drosophila TAF1 pre-mRNA in response to DNA damage. *Mol. Cell. Biol.* 26, 9256-9267. 10.1128/MCB.01125-06 17030624 PMC1698527

[DMM022590C36] Kawarai T., Pasco P. M. D., Teleg R. A., Kamada M., Sakai W., Shimozono K., Mizuguchi M., Tabuena D., Orlacchio A., Izumi Y. et al. (2013). Application of long-range polymerase chain reaction in the diagnosis of X-linked dystonia-parkinsonism. *Neurogenetics* 14, 167-169. 10.1007/s10048-013-0357-x 23435702

[DMM022590C37] Kupke K. G., Graeber M. B. and Müller U. (1992). Dystonia-parkinsonism syndrome (XDP) locus: flanking markers in Xq12-q21.1. *Am. J. Hum. Genet.* 50, 808-815. 1550125 PMC1682654

[DMM022590C38] Kwon Y.-J., Choi Y., Eo J., Noh Y.-N., Gim J.-A., Jung Y.-D., Lee J.-R. and Kim H.-S. (2013). Structure and expression analyses of SVA elements in relation to functional genes. *Genome Inform.* 11, 142-148. 10.5808/GI.2013.11.3.142 PMC3794087 24124410

[DMM022590C39] Lee W.-W. and Jeon B. S. (2014). Clinical spectrum of dopa-responsive dystonia and related disorders. *Curr. Neurol. Neurosci. Rep.* 14, 461 10.1007/s11910-014-0461-9 24844652 PMC4061475

[DMM022590C40] Lee L. V., Pascasio F. M., Fuentes F. D. and Viterbo G. H. (1976). Torsion dystonia in Panay, Philippines. *Adv. Neurol.* 14, 137-151. 941767

[DMM022590C41] Lee L. V., Kupke K. G., Caballar-Gonzaga F., Hebron-Ortiz M. and Müller U. (1991). The phenotype of the X-linked dystonia-parkinsonism syndrome. An assessment of 42 cases in the Philippines. *Medicine* 70, 179-187. 10.1097/00005792-199105000-00002 2030641

[DMM022590C42] Lee L. V., Munoz E. L., Tan K. T. and Reyes M. T. (2001). Sex linked recessive dystonia parkinsonism of Panay, Philippines (XDP). *Mol. Pathol.* 54, 362-368. 11724910 PMC1187125

[DMM022590C43] Lee L. V., Maranon E., Demaisip C., Peralta O., Borres-Icasiano R., Arancillo J., Rivera C., Munoz E., Tan K. and Reyes M. T. (2002). The natural history of sex-linked recessive dystonia parkinsonism of Panay, Philippines (XDP). *Parkinsonism Relat. Disord.* 9, 29-38. 10.1016/S1353-8020(02)00042-1 12217620

[DMM022590C44] Lee L. V., Rivera C., Teleg R. A., Dantes M. B., Pasco P. M. D., Jamora R. D. G., Arancillo J., Villareal-Jordan R. F., Rosales R. L., Demaisip C. et al. (2011). The unique phenomenology of sex-linked dystonia parkinsonism (XDP, DYT3, “Lubag”). *Int. J. Neurosci.* 121, 3-11. 10.3109/00207454.2010.526728 21047175

[DMM022590C45] Lohmann K. and Klein C. (2013). Genetics of dystonia: what's known? What's new? What's next? *Mov. Disord.* 28, 899-905. 10.1002/mds.25536 23893446

[DMM022590C46] Makino S., Kaji R., Ando S., Tomizawa M., Yasuno K., Goto S., Matsumoto S., Tabuena M. D., Maranon E., Dantes M. et al. (2007). Reduced neuron-specific expression of the TAF1 gene is associated with X-linked dystonia-parkinsonism. *Am. J. Hum. Genet.* 80, 393-406. 10.1086/512129 17273961 PMC1821114

[DMM022590C47] Marengo M. S. and Wassarman D. A. (2008). A DNA damage signal activates and derepresses exon inclusion in *Drosophila* TAF1 alternative splicing. *RNA* 14, 1681-1695. 10.1261/rna.1048808 18596254 PMC2491473

[DMM022590C48] Martin L., Grigoryan A., Wang D., Wang J., Breda L., Rivella S., Cardozo T. and Gardner L. B. (2014). Identification and characterization of small molecules that inhibit nonsense-mediated RNA decay and suppress nonsense p53 mutations. *Cancer Res.* 74, 3104-3113. 10.1158/0008-5472.CAN-13-2235 24662918 PMC4040335

[DMM022590C49] Müller U., Haberhausen G., Wagner T., Fairweather N. D., Chelly J. and Monaco A. P. (1994). DXS106 and DXS559 flank the X-linked dystonia-parkinsonism syndrome locus (DYT3). *Genomics* 23, 114-117. 10.1006/geno.1994.1465 7829058

[DMM022590C50] Nakamura K., Jeong S.-Y., Uchihara T., Anno M., Nagashima K., Nagashima T., Ikeda S.-I., Tsuji S. and Kanazawa I. (2001). SCA17, a novel autosomal dominant cerebellar ataxia caused by an expanded polyglutamine in TATA-binding protein. *Hum. Mol. Genet.* 10, 1441-1448. 10.1093/hmg/10.14.1441 11448935

[DMM022590C51] Németh A. H., Nolte D., Dunne E., Niemann S., Kostrzewa M., Peters U., Fraser E., Bochukova E., Butler R., Brown J. et al. (1999). Refined linkage disequilibrium and physical mapping of the gene locus for X-linked dystonia-parkinsonism (DYT3). *Genomics* 60, 320-329. 10.1006/geno.1999.5929 10493831

[DMM022590C52] Nolte D., Niemann S. and Muller U. (2003). Specific sequence changes in multiple transcript system DYT3 are associated with X-linked dystonia parkinsonism. *Proc. Natl. Acad. Sci. USA* 100, 10347-10352. 10.1073/pnas.1831949100 12928496 PMC193564

[DMM022590C53] Oyama G., Fernandez H. H., Foote K. D., Zeilman P., Hwynn N., Jacobson C. E. IV, Malaty I. A., Rodriguez R. L. and Okun M. S. (2010). Differential response of dystonia and parkinsonism following globus pallidus internus deep brain stimulation in X-linked dystonia-parkinsonism (Lubag). *Stereotact. Funct. Neurosurg.* 88, 329-333. 10.1159/000319961 20714213 PMC2969112

[DMM022590C54] Papai G., Weil P. A. and Schultz P. (2011). New insights into the function of transcription factor TFIID from recent structural studies. *Curr. Opin. Genet. Dev.* 21, 219-224. 10.1016/j.gde.2011.01.009 21420851 PMC3081712

[DMM022590C55] Ruppert S., Wang E. H. and Tjian R. (1993). Cloning and expression of human TAFII250: a TBP-associated factor implicated in cell-cycle regulation. *Nature* 362, 175-179. 10.1038/362175a0 7680771

[DMM022590C56] Sako W., Morigaki R., Kaji R., Tooyama I., Okita S., Kitazato K., Nagahiro S., Graybiel A. M. and Goto S. (2011). Identification and localization of a neuron-specific isoform of TAF1 in rat brain: implications for neuropathology of DYT3 dystonia. *Neuroscience* 189, 100-107. 10.1016/j.neuroscience.2011.05.031 21616129 PMC3150221

[DMM022590C57] Savage A. L., Bubb V. J., Breen G. and Quinn J. P. (2013). Characterisation of the potential function of SVA retrotransposons to modulate gene expression patterns. *BMC Evol. Biol.* 13, 101 10.1186/1471-2148-13-101 23692647 PMC3667099

[DMM022590C58] Schneider S. A. and Bhatia K. P. (2010). Rare causes of dystonia parkinsonism. *Curr. Neurol. Neurosci. Rep.* 10, 431-439. 10.1007/s11910-010-0136-0 20694531

[DMM022590C59] Schneider S. A., Bhatia K. P. and Hardy J. (2009). Complicated recessive dystonia Parkinsonism syndromes. *Mov. Disord.* 24, 490-499. 10.1002/mds.22314 19185014

[DMM022590C60] Sekiguchi T., Miyata T. and Nishimoto T. (1988). Molecular cloning of the cDNA of human X chromosomal gene (CCG1) which complements the temperature-sensitive G1 mutants, tsBN462 and ts13, of the BHK cell line. *EMBO J.* 7, 1683-1687. 3169001 10.1002/j.1460-2075.1988.tb02996.x PMC457153

[DMM022590C61] Sekiguchi T., Nohiro Y., Nakamura Y., Hisamoto N. and Nishimoto T. (1991). The human CCG1 gene, essential for progression of the G1 phase, encodes a 210-kilodalton nuclear DNA-binding protein. *Mol. Cell. Biol.* 11, 3317-3325. 10.1128/MCB.11.6.3317 2038334 PMC360184

[DMM022590C62] Stevanin G., Fujigasaki H., Lebre A.-S., Camuzat A., Jeannequin C., Dode C., Takahashi J., San C., Bellance R., Brice A. et al. (2003). Huntington's disease-like phenotype due to trinucleotide repeat expansions in the TBP and JPH3 genes. *Brain* 126, 1599-1603. 10.1093/brain/awg155 12805114

[DMM022590C63] Szpakowski S., Sun X., Lage J. M., Dyer A., Rubinstein J., Kowalski D., Sasaki C., Costa J. and Lizardi P. M. (2009). Loss of epigenetic silencing in tumors preferentially affects primate-specific retroelements. *Gene* 448, 151-167. 10.1016/j.gene.2009.08.006 19699787 PMC2783545

[DMM022590C64] Takahashi K. and Yamanaka S. (2006). Induction of pluripotent stem cells from mouse embryonic and adult fibroblast cultures by defined factors. *Cell* 126, 663-676. 10.1016/j.cell.2006.07.024 16904174

[DMM022590C65] Thomas M. C. and Chiang C.-M. (2006). The general transcription machinery and general cofactors. *Crit. Rev. Biochem. Mol. Biol.* 41, 105-178. 10.1080/10409230600648736 16858867

[DMM022590C67] Tsankov A. M., Akopian V., Pop R., Chetty S., Gifford C. A., Daheron L., Tsankova N. M. and Meissner A. (2015). A qPCR ScoreCard quantifies the differentiation potential of human pluripotent stem cells. *Nat. Biotech.* 33, 1182-1192. 10.1038/nbt.3387 PMC4636964 26501952

[DMM022590C68] Vogt J., Bengesser K., Claes K. B. M., Wimmer K., Mautner V.-F., van Minkelen R., Legius E., Brems H., Upadhyaya M., Högel J. et al. (2014). SVA retrotransposon insertion-associated deletion represents a novel mutational mechanism underlying large genomic copy number changes with non-recurrent breakpoints. *Genome Biol.* 15, R80 10.1186/gb-2014-15-6-r80 24958239 PMC4229983

[DMM022590C69] Wadia P. M., Lim S.-Y., Lozano A. M., Adams J. R., Poon Y.-Y., Torres Diaz C. and Moro E. (2010). Bilateral pallidal stimulation for x-linked dystonia parkinsonism. *Arch. Neurol.* 67, 1012-1015. 10.1001/archneurol.2010.187 20697054

[DMM022590C70] Wang H., Curran E. C., Hinds T. R., Wang E. H. and Zheng N. (2014). Crystal structure of a TAF1-TAF7 complex in human transcription factor IID reveals a promoter binding module. *Cell Res.* 24, 1433-1444. 10.1038/cr.2014.148 25412659 PMC4260347

[DMM022590C71] Waters C. H., Faust P. L., Powers J., Vinters H., Moskowitz C., Nygaard T., Hunt A. L. and Fahn S. (1993). Neuropathology of lubag (X-linked dystonia parkinsonism). *Mov. Disord.* 8, 387-390. 10.1002/mds.870080328 8341310

[DMM022590C72] Westenberger A., Rosales R. L., Heinitz S., Freimann K., Lee L. V., Jamora R. D., Ng A. R., Domingo A., Lohmann K., Walter U. et al. (2013). X-linked Dystonia-Parkinsonism manifesting in a female patient due to atypical turner syndrome. *Mov. Disord.* 28, 675-678. 10.1002/mds.25369 23389859

[DMM022590C73] Wilhelmsen K. C., Weeks D. E., Nygaard T. G., Moskowitz C. B., Rosales R. L., dela Paz D. C., Sobrevega E. E., Fahn S. and Gilliam T. C. (1991). Genetic mapping of “Lubag” (X-linked dystonia-parkinsonism) in a Filipino kindred to the pericentromeric region of the X chromosome. *Ann. Neurol.* 29, 124-131. 10.1002/ana.410290203 1672807

[DMM022590C74] Wray S., Self M., NINDS Parkinson's Disease iPSC Consortium, NINDS Huntington's Disease iPSC Consortium, NINDS ALS iPSC Consortium, Lewis P. A., Taanman J.-W., Ryan N. S., Mahoney C. J., Liang Y. et al. (2012). Creation of an open-access, mutation-defined fibroblast resource for neurological disease research. *PLoS ONE* 7, e43099 10.1371/journal.pone.0043099 22952635 PMC3428297

[DMM022590C75] Zech M., Castrop F., Schormair B., Jochim A., Wieland T., Gross N., Lichtner P., Peters A., Gieger C., Meitinger T. et al. (2014). DYT16 revisited: exome sequencing identifies PRKRA mutations in a European dystonia family. *Mov. Disord.* 29, 1504-1510. 10.1002/mds.25981 25142429

